# Wax-Based Sustained-Release Felodipine Oral Dosage Forms Manufactured Using Hot-Melt Extrusion and Their Resistance to Alcohol-Induced Dose Dumping

**DOI:** 10.3390/pharmaceutics17080955

**Published:** 2025-07-24

**Authors:** Gerard Sweeney, Dijia Liu, Taher Hatahet, David S. Jones, Shu Li, Gavin P. Andrews

**Affiliations:** 1 Pharmaceutical Engineering Group, School of Pharmacy, Queen’s University of Belfast, 97 Lisburn Road, Belfast BT9 7BL, UK; s1440802@qub.ac.uk (G.S.); dliu13@qub.ac.uk (D.L.); d.jones@qub.ac.uk (D.S.J.); s.li@qub.ac.uk (S.L.); 2 North Wales Medical School, Bangor University, Briganita Building, Sackville Road, Gwynedd, Bangor LL57 2DG, UK; t.hatahet@bangor.ac.uk

**Keywords:** hot-melt extrusion, wax, sustained release, dose dumping

## Abstract

**Background/Objectives:** Hot-melt extrusion (HME) has gained prominence for the manufacture of sustained-release oral dosage forms, yet the application of wax-based matrices and their resilience to alcohol-induced dose dumping (AIDD) remains underexplored. This study aimed to develop and characterise wax-based sustained-release felodipine formulations, with a particular focus on excipient functionality and robustness against AIDD. **Methods:** Felodipine sustained-release formulations were prepared via HME using Syncrowax HGLC as a thermally processable wax matrix. Microcrystalline cellulose (MCC) and lactose monohydrate were incorporated as functional fillers and processing aids. The influence of wax content and filler type on mechanical properties, wettability, and drug release behaviour was systematically evaluated. Ethanol susceptibility testing was conducted under simulated co-ingestion conditions (4%, 20%, and 40% *v*/*v* ethanol) to assess AIDD risk. **Results:** MCC-containing tablets demonstrated superior sustained-release characteristics over 24 h, showing better wettability and disintegration. In contrast, tablets formulated with lactose monohydrate remained structurally intact during dissolution, overly restricting drug release. This limitation was effectively addressed through granulation, where reduced particle size significantly improved surface accessibility, with 0.5–1 mm granules achieving a satisfactory release profile. Ethanol susceptibility testing revealed divergent behaviours between the two filler systems. Unexpectedly, MCC-containing tablets showed suppressed drug release in ethanolic media, likely resulting from inhibitory effect of ethanol on filler swelling and disintegration. Conversely, formulations containing lactose monohydrate retained their release performance in up to 20% *v*/*v* ethanol, with only high concentrations (40% *v*/*v*) compromising matrix drug-retaining functionality and leading to remarkably increased drug release. **Conclusions:** This study highlights the pivotal role of excipient type and constitutional ratios in engineering wax-based sustained-release formulations. It further contributes to the understanding of AIDD risk through in vitro assessment and offers a rational design strategy for robust, alcohol-resistant oral delivery systems for felodipine.

## 1. Introduction

Hot-melt extrusion (HME) has emerged as a compelling technique for continuous manufacturing, offering notable advantages over traditional batch processing. As a scalable and solvent-free manufacturing process, HME circumvents issues associated with residual organic solvents and time-consuming drying steps. More critically, it enables intensive mixing of drug and excipients under elevated temperatures and shear, facilitating the design of complex matrices with tailored release characteristics [1]. Felodipine, with a melting point of approximately 143–145 °C, has been widely explored in polymer-based amorphous solid dispersions via HME, primarily to enhance its solubility and dissolution rate. These efforts, however, typically demand high processing temperatures exceeding 130 °C [2,3]. By contrast, fewer studies have leveraged HME with the explicit objective of producing sustained-release systems for felodipine [4]. In a previous study comparing spray-drying and HME, HME-produced felodipine matrices exhibited a more prolonged release profile than their spray-dried counterparts, despite using the same matrix carrier. Although the evaluation was limited to a three-hour testing period, this may suggest the potential of HME as a manufacturing approach for extended-release felodipine dosage forms [5]. Subsequent research employed the rate-controlling polymer Kollidon^®^ SR via HME to achieve sustained drug release over 24 h [6].

While polymer-based sustained-release systems have demonstrated success, the high processing temperatures required for many polymer matrices present an industrial challenge. An alternative approach lies in the utilisation of wax-based carriers, which are inherently amenable to thermal processing and have been increasingly investigated in HME applications for sustained-release dosage forms [7]. These systems typically incorporate lipidic excipients such as natural waxes, hydrogenated vegetable oils, fatty acid esters, mono- and diacylglycerides, as well as long-chain triglycerides [8]. These materials offer low melting points, plasticising effects, and favourable thermal stability, facilitating extrusion at reduced temperatures and allowing the incorporation of thermally labile drugs. Additionally, their hydrophobicity enables the formation of diffusion-controlled matrices, which sustain drug release by modulating water ingress and drug diffusion pathways [7,8,9].

A recent investigation has demonstrated the feasibility of employing lipid-based excipients, specifically glyceryl monooleate, to develop HME-fabricated felodipine formulations with sustained-release characteristics. This formulation required the inclusion of a polymeric co-carrier, HPMC E5, to address processing challenges arising from the inherent stickiness and stiffness of glyceryl monooleate [10].

Syncrowax HGLC, the triglyceride ester of long-chain (C18-36) fatty acids, shows properties similar to Carnauba wax and a melting range of 60–70 °C [11], enabling low-temperature extrusion while maintaining a molten state throughout processing. Although this material has been investigated as a coating agent for beads via hot-melt coating, demonstrating favourable sustained-release properties, its potential as a matrix-forming carrier for the development of sustained-release formulations remains, to the best of our knowledge, entirely unexplored [12].

Nevertheless, like other wax-based systems, its low melt viscosity presents practical challenges within the extruder, often limiting drag flow and conveying efficiency. Instead of relying on polymeric carriers, the extrusion processability of waxy materials may also be optimised through the strategic incorporation of filler excipients [13]. Lactose monohydrate and microcrystalline cellulose (MCC), both well-established as conventional fillers in the pharmaceutical industry, are widely used in solid oral dosage forms due to their favourable compressibility and processibility. Their expanded application potential to HME has been explored and may potentially contribute to more cost-effective processing compared to polymer-based sustained-release systems. The incorporation of fillers can enhance material flow, stabilise the extrusion process, and ultimately contribute to desirable mechanical properties of extrudates for downstream steps, such as granulation and tableting [13,14,15].

Beyond facilitating extrusion, lactose monohydrate and MCC have been shown to exert distinct influences on drug release from HME-produced matrices. Lactose monohydrate, as a water-soluble excipient, dissolves upon contact with aqueous medium and generates aqueous channels within the matrix, enhancing medium ingress and facilitating effective drug release [14,16,17]. In contrast, MCC remains insoluble but swells upon hydration, fragmenting the dosage form and increasing surface exposure. Its internal porous structure further contributes to media penetration and desired drug release [15,18]. These functional differences have been exploited in both immediate- and sustained-release systems produced via HME, affecting matrix erosion behaviour and drug release kinetics.

Another critical aspect impacting the performance of sustained-release formulations is the risk of alcohol-induced dose dumping (AIDD), a phenomenon characterised by the unintended, rapid drug release from modified-release systems following co-ingestion of alcohol [19]. Despite regulatory efforts and technological progress, the development of oral sustained-release systems with robustness against AIDD remains inherently challenging. Contributing factors include the complicated manufacturing requirements, such as the need for additional coating or casting steps, the limited availability of well-established commercial alcohol-resistant technologies, and the constraints imposed by excipient selection, which often demands a delicate balance of functionality and processability [20,21]. This issue is of particular concern for chronic medications such as felodipine, which require prolonged, controlled release to maintain steady-state levels. Such formulations typically desire a drug delivery over 12–24 h and therefore contain a larger unit of dose than conventional immediate-release products, which are often administered every 4–6 h. Co-administration of alcohol-containing food or beverages can significantly alter drug solubility, impair excipient performance, compromise matrix integrity, and thus disrupt the intended release-controlling mechanisms. These effects collectively increase the risk of overdose, resulting in systemic toxicity and exacerbated side effects [22]. A pertinent example is Palladone™, an extended-release formulation of hydromorphone, which was withdrawn from the market in 2005 due to fatal interaction with alcohol [23]. Although in vivo pharmacokinetic studies could theoretically evaluate AIDD risk, they are often impractical and ethically problematic. Therefore, it becomes imperative to assess the resistance of controlled-release formulations to alcohol-mediated alterations during in vitro drug release.

In this context, the present study aimed to investigate the influence of wax content and filler type on the development of oral sustained-release felodipine formulation manufactured via HME. The resulting tablets and granules were evaluated for their drug release behaviour, and those deemed appropriate were further examined in alcohol-containing dissolution media to assess formulation resilience against AIDD.

## 2. Experimental Section

### 2.1. Materials

Felodipine was obtained from AstraZeneca (Batch No. 0000272586, Gothenburg, Sweden). Syncrowax HGLC was supplied by Croda (Product Code: SW42815, Yorkshire, UK). Microcrystalline cellulose (Avicel^®^ PH-101, Batch No. BCCJ6248), α-lactose monohydrate (≥99%, Batch No. 0000456598), ethanol (≥99.50%), hydrochloric acid (37%) sodium chloride (≥99.0%), sodium phosphate dibasic (≥99.0%) and sodium phosphate dibasic (≥99.0%) were obtained from Sigma-Aldrich (Merck, Darmstadt, Germany). All other chemicals used were of analytical grade and purchased from BDH Laboratory supplies (Dorset, UK).

### 2.2. Methods

#### 2.2.1. Preparation of Felodipine Granules by Hot-Melt Extrusion

Formulations were prepared containing 5% *w*/*w* felodipine. The appropriate quantities of felodipine, filler (either lactose monohydrate or MCC), and Syncrowax HGLC were accurately weighed, premixed, and fed into a co-rotating twin-screw extruder (Minilab, Thermo Electron Corporation, Karlsruhe, Germany) equipped with a 2 mm diameter round die. Extrusion was conducted under the operating conditions detailed in Table 1. The resulting rod-shaped extrudates were manually milled using a pestle and mortar, then sieved to obtain granules within two particle size ranges: 0.5–1 mm and 1–2 mm. A portion of the granules was retained for direct dissolution testing to enable comparison with the corresponding tablet formulations.

Powder X-ray diffraction (PXRD) patterns were collected using a PANalytical X’Pert PRO Diffractometer controlled via the X’Pert Data Collector HighScore (PANalytical, Almelo, the Netherlands) with Cu Kα radiation (wavelength = 1.5406 À) generated from a copper source operating at a power level of 40 kV. The test samples were packed onto graphite sample holders and were scanned from 3° to 40° 2θ at a scan rate of 0.0423° 2θ per minute. The time per step was 50 s and the step size was 0.0167° 2θ.

#### 2.2.2. Preparation of Compressed Tablets

Tablets were prepared by compressing 400 mg of granules using a hydraulic press (Specac Ltd., Orpington, UK) at a pressure of 2 tonnes for 30 s. Tablet thickness was measured using a Mitutoyo 293-831-30 digital micrometer (Sheffield, UK). Compression was performed using a 13 mm diameter die, enabling the calculation of tablet volume and, subsequently, density (D) using the following equation [24]:

$$D\;({g/\mathrm{cm}}^{3})\;=\;\frac{w}{{\left(d/2\right)}^{2}\times \pi \times h\;}$$

where *w* is the mass of the tablet (g), *d* is the diameter of the tablet (cm), and *h* is the tablet thickness (cm). All reported values represent the mean ± standard deviation (SD) of six replicates (n = 6).

#### 2.2.3. Tensile Strength and Mechanical Strength of Tablets

The breaking force of the tablets was determined using a 5Y Hardness Tester (Copley Scientific, Nottingham, UK). Results are reported as the mean ± SD of five replicates (n = 5). The measured breaking force was subsequently used to calculate the tensile strength (*σ_t_*) of the tablets using the following equation [25]:

$$\sigma_{t}\left(MPa\right)=\;\frac{2F}{\pi \;\times d\;\times t\;}$$

where *F* is the force (N) required to fracture the tablet, *d* is the diameter (mm), and *t* is the thickness (mm) of the tablet.

The mechanical strength of the tablets was further assessed using a Tablet Friability Tester FR 1000 (1 drum, fixed speed; Copley Scientific, Nottingham, UK), following the European Pharmacopoeia method for friability testing in accordance with European Pharmacopoeia (Ph. Eur.2.9.7) [26]. Briefly, a specified number of tablets (in accordance with compendial requirements) were accurately weighed and placed in a rotating drum with an internal radius of 80 mm, extending from the centre to the outer wall. The drum was rotated 100 times at a fixed speed of 25 ± 1 rpm. During rotation, tablets tumbled and impacted the drum wall or each other. After the test, tablets were dedusted and reweighed if none were cracked, split, or broken. Friability was calculated as the percentage loss in tablet mass using the equation below:

$$\mathrm{Friability}\;(\%)=\;\frac{\mathrm{Inital}\;\mathrm{Mass}-\mathrm{Final}\;\mathrm{Mass}}{\mathrm{Intial}\;\mathrm{Mass}}\;\times 100$$

#### 2.2.4. Contact Angle Measurement

Contact angle measurements were performed using a First Ten Angstroms FTA200 dynamic contact angle analyser equipped with a video capture system (First Ten Angstroms, Inc., Portsmouth, VA, USA). Image acquisition and analysis were conducted using FTA32 video software 2.0 (First Ten Angstroms, Inc., Portsmouth, VA, USA). A 5 μL droplet of distilled water was precisely dispensed onto the tablet surface using a calibrated microsyringe. Each droplet was analysed immediately upon deposition, with image acquisition and angle measurement completed within 10 s. The contact angle was determined by measuring the angle between the baseline of the droplet and the tangent at the droplet boundary. Measurements were taken on both sides of each droplet, and the average value was reported. A minimum of ten measurements were conducted for each tablet formulation (n = 10).

#### 2.2.5. In Vitro Dissolution Testing

The in vitro dissolution behaviour of the formulations was evaluated in triplicate using the basket method (100 rpm) with a Caleva 7ST dissolution bath (GB Caleva Ltd., Shaftesbury, UK) maintained at 37 °C. Tablets or equivalent quantities of granules were introduced into 500 mL of pH 6.8 phosphate-buffered saline (PBS) containing 2% *w*/*w* polysorbate 20 [27]. This medium was selected to ensure sink conditions for felodipine. At predetermined time intervals, 5 mL aliquots were withdrawn from each vessel and filtered through a 0.45 µm cellulose acetate filter (Nalgene Labware, Rochester, NY, USA). An equivalent volume of fresh dissolution medium was replaced after each sampling.

Felodipine concentration in each sample was quantified using a Cary 50 UV–VIS spectrophotometer (Varian Ltd., Oxford, UK) at 362 nm (λ_max_). Quantification was based on a validated calibration curve (R^2^ ≥ 0.999), which was linear over the concentration range of 1.0–50.0 µg/mL. Samples exceeding this range were diluted appropriately with fresh medium. No interference from excipients at 362 nm was observed. The percentage of felodipine dissolved at each time point was calculated (n = 3) and plotted against time.

To further investigate the mechanism of drug release, dissolution data were fitted to the Korsmeyer–Peppas (Power Law) model [28]. In accordance with standard practice, only the portion of the dissolution curve corresponding to a fractional release (*Q_t_*/*Q_∞_*) of ≤0.6 was considered for model fitting. Model parameters were calculated using Microsoft Excel (Version 2505, Microsoft Corp., Redmond, WA, USA). The applied model is expressed as follows:

$$\frac{Q_{t}}{Q_{\infty }}=Kt^{n}$$

where *Q_t_* is the cumulative amount released by time *t*, *Q_∞_* is the maximum (theoretical) amount released at infinite time, *t* is the time, *n* is the release exponent, and *K* is the kinetic (rate) constant.

Although initially developed for immediate-release dosage forms to assess bioequivalence, the similarity factor (f_2_) is also applicable to modified-release formulations [29]. The comparison between the dissolution profiles of interest was conducted based on the following equation [30]:

$$f_{2}\;=\;50\times log\{\;{\left[1\;+\;\frac{1}{n}\sum_{t=1}^{n}{\left(R_{t}-T_{t}\right)}^{2}\right]}^{-0.5}\times 100\;\}$$

where *n* is the number of sampling time points, *R_t_* and *T_t_* represent the percentage of drug dissolved at each time point *t* for the reference and test formulations, respectively. A value between 50 and 100 is generally accepted as indicative of similarity between the dissolution profiles.

#### 2.2.6. Effect of Ethanol on Felodipine Solubility

To evaluate the impact of ethanol on the solubility of felodipine in the dissolution medium, excess drug was added to vials containing 10 mL of pH 6.5 PBS supplemented with 2% *w*/*w* polysorbate 20. Ethanol was incorporated into the medium at concentrations of 4%, 20%, and 40% (*v*/*v*) by replacing the corresponding volume of the aqueous phase. The vials were incubated in an ISF-7100 Incubated Shaker (Jeio Tech, Seoul, Republic of Korea) at 37 °C and agitated at 60 rpm for 24 h. Following incubation, the solutions were filtered through a 0.45 µm cellulose acetate filter, diluted with fresh media as required, and analysed for felodipine content using UV–VIS spectroscopy.

#### 2.2.7. Effect of Ethanol on Felodipine Release Behaviour

The influence of ethanol on the drug release behaviour of selected formulations was assessed by incorporating ethanol into the dissolution media at concentrations of 4%, 20%, and 40% (*v*/*v*), consistent with levels reported to simulate alcohol ingestion scenarios [19,20]. Dissolution testing was performed using a two-stage protocol designed to reflect physiologically relevant conditions. Initially, tablets or granules were placed in 500 mL of acidic medium (0.1 N HCl supplemented with 2% *w*/*w* polysorbate 20) containing the designated ethanol concentrations for a duration of 2 h. Following this, the dissolution medium was completely replaced with fresh pH 6.8 PBS containing the corresponding ethanol concentrations, and the dissolution study continued for the remaining period.

The concentration of felodipine in the sampled aliquots was determined by UV–VIS spectrophotometry. Prior validation confirmed that ethanol did not interfere with absorbance readings at 362 nm and established calibration curves. The f_2_ was calculated to assess the influence of ethanol-containing media on drug release profiles, in accordance with FDA recommendation [20].

#### 2.2.8. Disintegration Testing

Tablet disintegration was assessed using a DTG100 disintegration tester (Copley Scientific, Nottingham, UK) as per Ph. Eur. method [31]. The test medium was maintained at 37 °C using a thermostatically controlled water bath. Six tablets were placed into the individual holders of the disintegration basket and secured with plastic discs. The basket was subjected to a repetitive up-and-down motion. The disintegration time was recorded as the point at which all six tablets had completely disintegrated, leaving no palpable core.

#### 2.2.9. Statistical Analysis

The effects of particle size and filler content on the physical properties of tablets, as well as the felodipine release rate in the presence and absence of ethanol, were statistically evaluated using Microsoft Excel 2013 with the Analysis ToolPak add-in (Microsoft Corporation, Redmond, WA, USA). One-way analysis of variance (ANOVA) followed by Tukey’s post hoc test was employed to determine significant differences between groups. Where applicable, group means were compared using Student’s *t*-test. A *p*-value < 0.05 was considered statistically significant.

## 3. Results and Discussion

### 3.1. HME Manufacturing of Wax-Based Granules

The materials employed in this study, felodipine, Syncrowax HGLC, MCC, and lactose monohydrate, have all been previously utilised in HME processing and demonstrated sufficient thermal stability at the intended processing temperature (≤70 °C) [12,32,33,34,35].

Because the extrusion was performed well below the melting point of felodipine, physical state alteration such as amorphisation was not expected or intended, given the focus of this study lies in sustained-release formulation development rather than solubility enhancement. For completeness, preliminary PXRD analysis of the prepared extrudates was conducted, with characteristic diffraction peaks of felodipine clearly observed (Figure S1, Supplementary Materials), confirming its crystalline nature post-processing. Although slight variations in peak intensity may suggest differing apparent crystallinity between formulations, it should be noted that the low drug loading (5% *w*/*w*) renders quantitative interpretation unreliable [36].

As summarised in Table 1, extrusion parameters varied with wax content and filler type, as the primary objective was to yield solid extrudates suitable for downstream steps and to ensure stable processing. Increasing the wax content facilitated extrusion at lower processing temperatures, due to the higher proportion of molten phase within the formulation, which improved material flow and softened the matrix. This effect was observed consistently across both filler systems. However, noticeable differences in processability emerged when comparing fillers at equivalent wax content. Formulations incorporating lactose monohydrate were found to be more extrudable at lower temperatures, allowing a reduction in screw speed from 100 rpm to 80 rpm. In contrast, MCC-containing formulations required higher extrusion temperatures to achieve a consistent output and acceptable extrudate quality.

These differences in processability may be influenced by several interrelated material properties. First, the markedly lower bulk density of MCC (0.2–0.3 g/cm^3^) [37] compared to lactose monohydrate (0.7–0.9 g/cm^3^) [38] could result in a looser powder bed within the extruder, potentially leading to suboptimal barrel fill and diminished drag flow. This may render the material more susceptible to inconsistent melt formation, necessitating more aggressive agitation applied to achieve stable material conveyance. Moreover, at a fixed mass feed rate, the increased volumetric occupancy associated with low-density MCC could reduce shear efficiency within the barrel, and therefore, a reduced screw speed may not be viable [13,39,40]. Second, the lower thermal conductivity of MCC relative to lactose monohydrate may further hinder heat transfer within the barrel, delaying the effective melting of wax matrices [41,42]. To compensate, higher barrel temperatures would be required to promote sufficient molten phase development for smooth and continuous extrusion.

### 3.2. Mechanical Properties of Tablets

The prepared extrudates were readily compressed into tablets without the additional need for auxiliary tableting excipients. This favourable compressibility can be attributed to two factors. First, tablet compaction was performed manually using a hydraulic press, where material flowability is not a critical constraint. Second, the wax-based matrix produced via HME likely exhibited inherent binding properties, facilitating direct compression. The ability of lipidic and waxy materials to act as meltable binders in molten-based processing has been well reported and may explain the successful direct compression achieved in this study [43,44,45].

Tablet mechanical strength was evaluated from two perspectives: fracture resistance (tensile strength) and attrition resistance (friability) [46]. The physical properties of the prepared tablets are summarised in Table 2. For formulations containing either type of filler, increasing the wax content led to a statistically significant reduction in tensile strength (*p* < 0.05), likely due to the inherently brittle nature of the wax matrix upon colling [47]. Tablets formulated with lactose exhibited lower tensile strength than their MCC counterparts at equivalent wax levels, indicating a comparatively softer structure. In pharmaceutical manufacturing, a tensile strength range of 1–3 MPa is generally recognised as highly relevant for industrial practice. In this study, the formulated tablets exhibited tensile strengths between 1.0 and 1.5 MPa. Such hardness is not too challenging for tablet disintegration following administration, which is favourable; however, the relatively low tensile strength may predispose the tablets to mechanical damage, such as breakage or fragmentation [48,49]. To further evaluate the mechanical robustness of the formulations, friability testing was performed. All tablets demonstrated friability values below the USP limit of 1.0% *w*/*w* [50], confirming their durability for routine handling, packaging, and storage.

**Figure 1:**
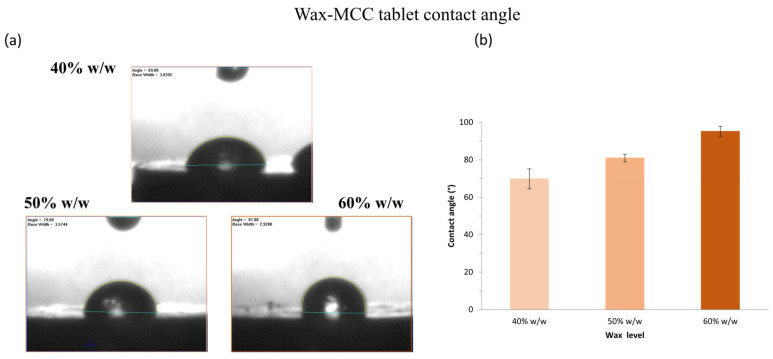
(**a**) Representative contact angle images of MCC tablets containing 40% *w*/*w*, 50% *w*/*w* and 60% *w*/*w* wax. (**b**) Effect of wax level on the contact angle of MCC tablets containing 40% *w*/*w*, 50% *w*/*w* and 60% *w*/*w* wax. Each value is the average ± SD of at least ten replicates (n = 10).

**Figure 2:**
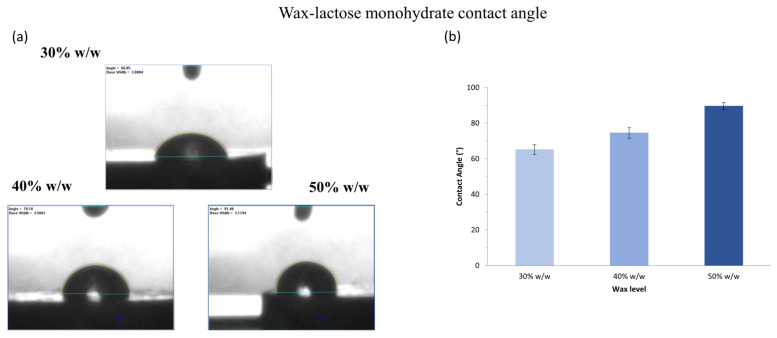
(**a**) Representative contact angle images of lactose monohydrate tablets containing 30% *w*/*w*, 40% *w*/*w* and 50% *w*/*w* wax. (**b**) Effect of wax level on the contact angle of lactose monohydrate tablets containing 30% *w*/*w*, 40% *w*/*w* and 50% *w*/*w* wax. Each value is the average ± SD of at least ten replicates (n = 10).

Tablet wettability was assessed using the sessile drop method to measure static contact angles. Representative images and results for formulations containing varying wax levels are shown in Figure 1 and Figure 2 for tablets prepared with MCC and lactose, respectively. Contact angle serves as an indicator of surface wettability, reflecting the ability of materials to absorb water and initiate internal wicking, key factors influencing dissolution performance [51]. Across all formulations, contact angles exceeded 65°, indicating the hydrophobic character of the wax-based matrices. As expected, increasing wax content significantly elevated the contact angle (*p* < 0.05), consistent with the greater hydrophobicity imparted by the lipidic excipient. At equivalent wax levels, MCC-containing tablets exhibited significantly lower contact angles than those containing lactose, suggesting superior wettability. This may be associated with their rough and porous surface, which enhances water affinity and penetration, likely contributing to faster disintegration and more efficient drug release [52].

### 3.3. In Vitro Dissolution Studies

#### 3.3.1. Drug Release Profiles of Formulations with MCC

**Figure 3:**
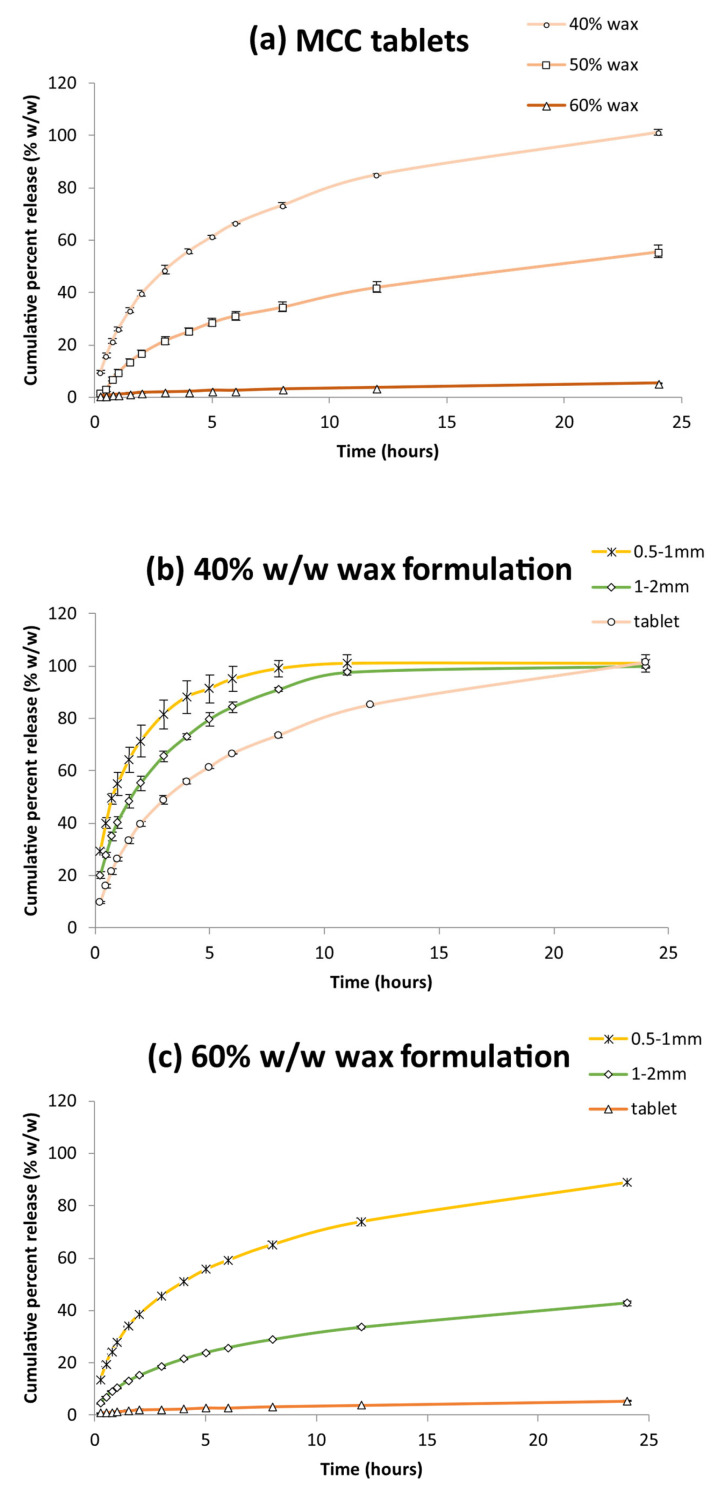
(**a**) Felodipine release from MCC tablets containing 40% *w*/*w*, 50% *w*/*w* and 60% *w*/*w* wax. (**b**) Felodipine release from 40% *w*/*w* wax formulations; 0.5–1 mm granules, 1–2 mm granules and tablet (**c**) Felodipine release from 60% *w*/*w* wax formulations; 0.5–1 mm granules, 1–2 mm granules and tablet. Each value is the average of three replicates (n = 3) ± SD and the coefficient of variation in all cases was less than 10%.

The dissolution profiles of wax-based felodipine tablets formulated with MCC are illustrated in Figure 3a. The impact of wax content on drug release was assessed using the similarity factor (f_2_), a statistical tool used to evaluate the degree of similarity between dissolution profiles. Increasing the wax concentration from 40% to 60% *w*/*w* substantially suppressed the release of felodipine (f_2_ = 26.8 for 40% vs. 50% and f_2_ = 30.4 for 40% vs. 60%, *p* < 0.05). At the highest wax loading (60% *w*/*w*), only ~5% of the drug was released over 24 h, in stark contrast to the complete release from 40% wax-based tablets. These results suggest that 40% *w*/*w* represents an optimal wax content when combined with MCC, offering an appreciable sustained-release effect without compromising the overall release extent.

This pronounced reduction in drug release with increasing wax content is likely due to its hydrophobic nature of felodipine (log P = 3.86) [53], which favours partitioning into the hydrophobic wax matrix rather than the aqueous dissolution medium. As the wax content increases, this affinity restricts the diffusion of drug molecules into the surrounding fluid, attenuating release rate and extent.

Beyond wax content, the dosage form (specifically, tablet versus granules) was also found to remarkably influence the release behaviour. For the 40% *w*/*w* wax formulation (Figure 3b), felodipine granules notably accelerated drug release compared to tablets, with f_2_ values of 41.6 and 42.2 for 1–2 mm and 0.5–1 mm granules, respectively. Such acceleration is attributable to the increased surface area available for dissolution, which facilitates more rapid water interaction and drug diffusion from the wax base [9]. Despite this, since all formulations achieved complete release, the effect of granulation at this wax level appeared to enhance release kinetics rather than the extent of drug release.

A similar trend was observed with the 60% *w*/*w* wax formulation (Figure 3c), where particle size reduction from tablets to granules also increased the release rate. f_2_ values decreased to 35.2 for 1–2 mm granules and further to 27.3 for 0.5–1 mm granules. Notably, in this case, the enhancement in release was not limited to kinetics; the achieved drug concentration by the end of testing increased dramatically, with the smallest granules (0.5–1 mm) approaching nearly 90% release, a 16-fold increase compared to the tablets.

This contrast holds critical implications for formulation design. In low-wax systems (e.g., 40% *w*/*w*), which are already capable of full drug release, granulation primarily increases the release rate and may undermine sustained-release goals by promoting premature drug delivery. Conversely, in high-wax systems (e.g., 60% *w*/*w*), where release is otherwise overly restricted, granulation plays a functionally transformative role by alleviating the retentive effect of wax-rich sustained-release matrices and enabling more effective drug liberation.

#### 3.3.2. Drug Release Profiles of Formulations with Lactose Monohydrate

**Figure 4:**
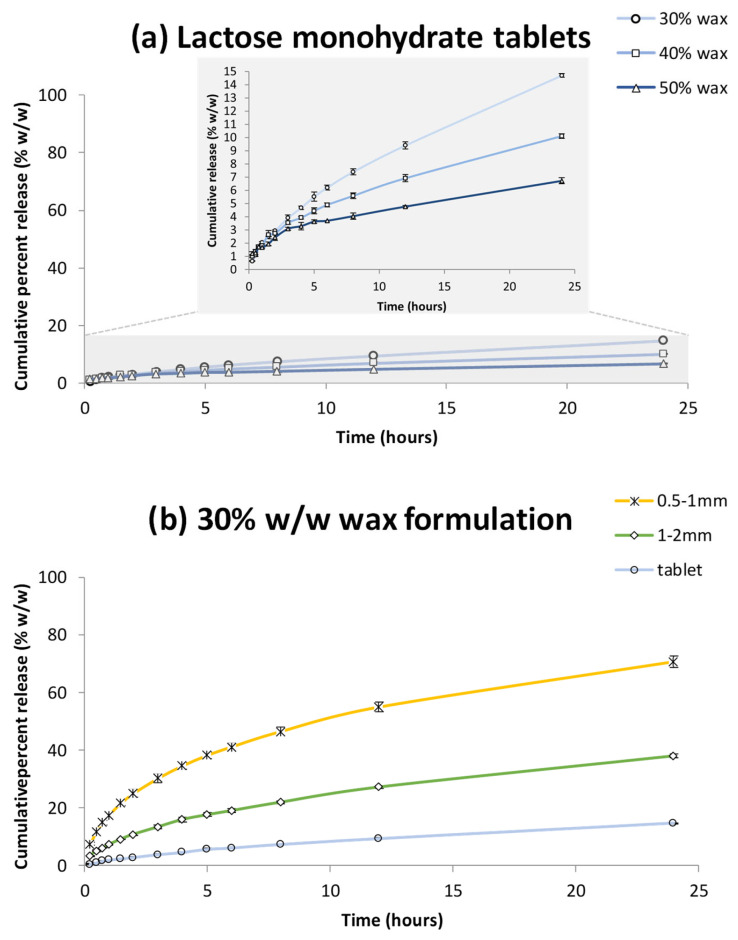
(**a**) Felodipine release from Lactose monohydrate tablets containing 30% *w*/*w*, 40% *w*/*w* and 50% *w*/*w* wax; a magnified inset illustrates the release profiles for improved clarity. (**b**) Felodipine release from 30% *w*/*w* wax formulations; 0.5–1 mm granules, 1–2 mm granules and tablet. Each value is the average of three replicates (n = 3) ± SD and the coefficient of variation in all cases is less than 10%.

The dissolution behaviour of felodipine tablets formulated with lactose monohydrate is illustrated in Figure 4. Across the tested wax concentrations (30% to 50% *w*/*w*), no statistically significant differences were observed in the drug release profiles (f_2_ = 75.6 for 30% vs. 50% *w*/*w* wax, *p* > 0.05; Figure 4a). Throughout the testing period, tablets remained structurally intact, with no evident disintegration. This limited release performance is presumably linked to the dependence on direct surface contact with water, which appears to be critical for drug dissolution in the developed wax-based systems [9,54]. As effective drug release is largely governed by increased surface exposure following disintegration, the absence of this process led to minimal drug release.

Such dependency is further evidenced by the comparison between tablets formulated with MCC and lactose monohydrate. At equivalent wax levels, lactose-containing tablets released significantly less felodipine. Specifically, for formulations based on 40% and 50% *w*/*w* wax, f_2_ values comparing MCC and lactose tablets were 16.6 and 31.1, respectively (*p* < 0.05), highlighting substantial divergence in dissolution behaviour. While MCC, a well-known disintegrant, facilitated rapid disintegration upon hydration and thereby generated extensive surface area for dissolution [55], lactose-containing tablets relied merely on the gradual dissolution of lactose monohydrate to create aqueous channels, hence resulting in an inherently slower and less efficient process for enhancing surface area [16].

Building on these insights, the 30% *w*/*w* wax formulations using lactose monohydrate as the filler, superior to the other wax contents, was further evaluated in granulated forms (Figure 4b). Drug release was markedly enhanced in both granule size fractions, with f_2_ = 46.4 between tablets and 1–2 mm granules, and f_2_ = 36.3 between 1–2 mm and 0.5–1 mm granules (*p* < 0.05). The finest granules (0.5–1 mm) exhibited the most favourable release profile, achieving a sustained, prolonged release over 24 h and reaching a final drug release of approximately 70%, a nearly fivefold increase compared to the 15% observed in compressed tablets.

#### 3.3.3. Release Kinetics: Korsmeyer–Peppas Model Analysis

To further understand the drug release mechanisms from wax-based felodipine tablets, the dissolution data were fitted to the Korsmeyer–Peppas model (Table 3). The high coefficient of determination (R^2^ > 0.96) across all applicable profiles confirms an excellent fit, supporting the appropriateness of this model for describing release kinetics in these systems [56]. The fitted release curves, along with the corresponding equations and model parameters for each dataset, are presented in Figure S2.

Across both filler types, the release exponent (*n*) decreased progressively with increasing wax concentration, revealing a shift from anomalous (non-Fickian) transport towards a diffusion-controlled (Fickian) mechanism [56]. This suggests that at higher wax content, which restricts water ingress and retards matrix erosion, drug release becomes more dominated by diffusion through the dense hydrophobic matrix [54]. At comparable wax levels, tablets containing MCC exhibited higher n values than those with lactose monohydrate, highlighting the role of swelling and disintegration behaviour of MCC in modulating the release mechanism.

These findings corroborate earlier dissolution data and clarify the mechanistic contributions of both wax content and filler type. While wax concentration primarily governs the extent to which diffusion dominates, the choice of filler plays a crucial supportive role in facilitating water interaction and internal matrix disruption. MCC promotes tablet swelling and disintegration, enhancing water penetration and drug release from tablets. Conversely, formulations with lactose monohydrate preserve their structural integrity, thereby limiting water access and confining drug release to diffusion through the intact wax matrix [57,58].

Granule formulations subjected to dissolution testing were also fitted to the Korsmeyer–Peppas model, with the corresponding results presented in Table 4. Among the tested samples, all the 60% wax formulations filled with MCC, including tablet and granules, exhibited n values between 0.45 and 0.46, indicative of consistent Fickian diffusion. For the other two tablet formulations, 30% wax with lactose monohydrate and 40% wax with MCC, an apparent reduction in n values was observed for the corresponding granulated formulations. Specifically, n values decreased from above 0.6 for the tablets to 0.46–0.49 for MCC-containing granules and 0.51–0.53 for granules with lactose monohydrate. This decrease may be associated with the absence of an intermediate disintegration or swelling step. In tablet matrices, hydration and subsequent swelling often give rise to anomalous transport mechanisms, whereas in granules, already present in discrete form, the release is more directly governed by diffusion through the wax matrix [59]. These findings suggest the influence of dosage form geometry and compactness on the prevailing drug release mechanism, although further rationalised investigation is needed to substantiate this interpretation.

### 3.4. Effect of Ethanol on Felodipine Release Behaviours

AIDD poses a recognised safety concern for controlled-release formulations, particularly those employing lipid-based or hydrophobic matrices. Ethanol can act as a potent co-solvent, enhancing the solubility of drug and excipients and obstructing the release-controlling function of the matrix. This risk is further amplified in sustained-release systems, which typically contain a higher drug load as a reservoir to fulfil prolonged therapeutic action. Hence, AIDD may result in abrupt, uncontrolled plasma concentrations and pose a significant risk of overdose and systemic toxicity [60].

Although calcium channel blockers, including felodipine, are uncommon in overdose, an upward trend in reported cases has been observed in recent years, with the median annual incidence during 2014–2023 tripling compared to the preceding decade [61]. Notably, alcohol has been identified as one of the most frequently co-consumed substances among patients receiving this class of medication [62]. Given these concerns, it is crucial to evaluate the ethanol responsiveness of the wax-based felodipine matrix developed in this study. The following sections explore the solubility of felodipine in ethanol-containing media and investigate the in vitro release performance of the developed formulations under conditions related to the concomitant intake of alcohol.

It is also important to recognise that the gastrointestinal tract introduces additional physiological variables not captured by the dissolution testing reported herein, such as the presence of bile salts and digestive enzymes, particularly lipases. While traditionally regarded as chemically inert during processing, the susceptibility of wax-based excipients to lipid digestion has garnered increasing attention in the evaluation of formulation performance. In the presence of bile salts and lipolytic enzymes, the wax matrix may undergo emulsification and enzymatic degradation, significantly accelerating carrier erosion compared to simple buffered conditions. This process can, in turn, result in markedly enhanced drug release, premature drug exposure, and consequently, an exaggerated risk of dose dumping. Therefore, while the current study primarily addresses ethanol-induced effects, future investigations employing biorelevant dissolution media containing bile components and digestive enzymes are warranted to comprehensively assess the robustness and in vivo relevance of the developed sustained-release formulations [63,64,65].

#### 3.4.1. Felodipine Solubility in Ethanol-Containing Media

Felodipine has been reported to show ethanol-sensitive solubility and demonstrate susceptibility to ethanol co-ingestion in both in silico simulations and in vivo studies [66,67]. Although the extent of solubility increasement and associated pharmacokinetic alterations in the presence of ethanol varies considerably across reports, evaluating the ethanol resistance of felodipine oral formulations is still essential. To assess this potential risk, felodipine solubility was first examined in the previously used dissolution medium supplemented with increasing concentrations of ethanol (4%, 20%, and 40% *v*/*v*), simulating levels typically encountered in alcoholic beverages [68].

As shown in Table 5, felodipine solubility remained largely unchanged from 0% to 20% ethanol. However, a marked increase in solubility, approximately fourfold, was observed at 40% ethanol, reflecting a pronounced co-solvent effect at higher ethanol concentrations. This highlights the relevance of evaluating drug release behaviour under ethanol-containing conditions for sustained-release formulations of felodipine. Nevertheless, since sink conditions were maintained throughout the dissolution testing, the altered solubility is not expected to be a limiting factor in this study. Still, attention should be paid to the ethanol-enhanced solubility of felodipine, which could become pharmacologically significant under in vivo conditions.

#### 3.4.2. Ethanol Impact on Wax–Lactose Monohydrate Granules

Based on earlier findings, lactose-based tablets exhibited minimal drug release and were thus excluded from this stage of ethanol-impact testing. Instead, 0.5–1 mm granules containing 30% *w*/*w* wax and lactose monohydrate, which showed superior release performance, were evaluated further.

**Figure 5:**
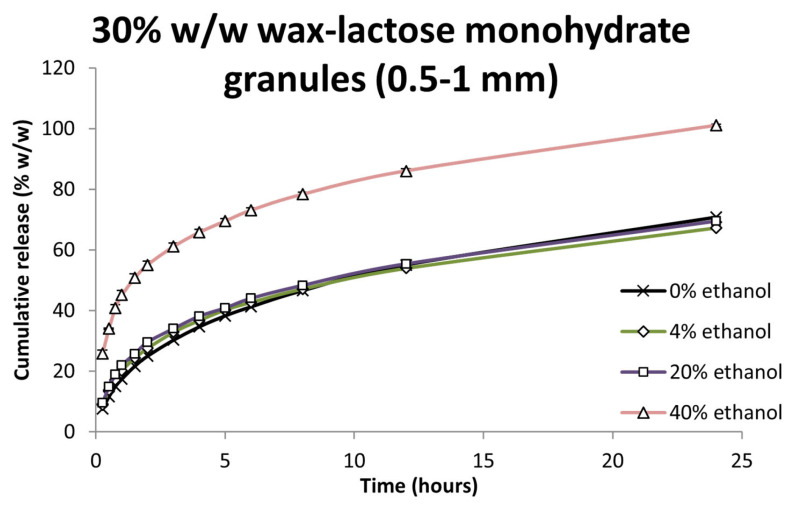
Felodipine dissolution from 30% *w*/*w* wax–lactose monohydrate granules size (0.5–1 mm) in 0%, 4%, 20% and 40% *v*/*v* ethanol. Each value is the average of three replicates (n = 3) ± SD and the coefficient of variation in all cases is less than 10%.

As illustrated in Figure 5, the inclusion of ethanol at concentrations up to 20% *v*/*v* had no statistically significant effect on drug release compared to ethanol-free media (f_2_ = 73.9, *p* > 0.05). This indicates that the formulation retained its release-controlling properties and was resistant to moderate levels of ethanol exposure. However, when the ethanol concentration was increased to 40% *v*/*v*, drug release was markedly enhanced, achieving near-complete release by the end of the study period (f_2_ = 29.5, *p* < 0.05), signifying a loss of the original release-retarding effect of the matrix.

This ethanol-induced acceleration of drug release may be associated with the weakened ability of wax to retain the drug and thereby enhancing dissolution in high-ethanol environments. The absence of such an effect at lower ethanol levels is plausibly due to the insolubility of lactose monohydrate in ethanol. In aqueous environments, lactose dissolves readily, contributing to the formation of internal networks for water ingress and drug release. Ethanol, by contrast, is a poor solvent for lactose [69,70], limiting its dissolution and thus impeding the development of aqueous pathways through the matrix. As a result, the wax structure remains largely intact, continuing to hinder drug release. At 40% ethanol, however, this delicate balance appears to be significantly altered, resulting in a breakdown of the original release-retarding mechanism.

#### 3.4.3. Ethanol Impact on Wax–MCC Tablets

**Figure 6:**
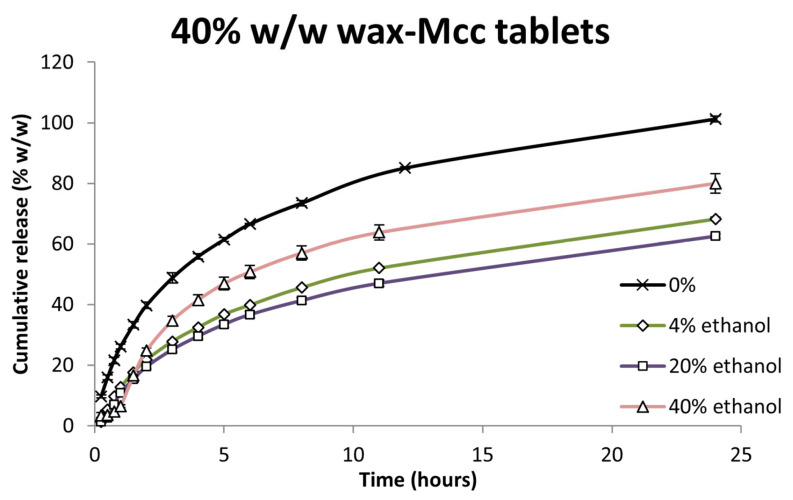
Felodipine dissolution from 40% *w*/*w* wax–MCC tablets in 0%, 4%, 20% and 40% *v*/*v* ethanol. Each value is the average of three replicates (n = 3) ± SD and the coefficient of variation in all cases is less than 10%.

Figure 6 depicts the dissolution profiles of MCC-containing tablets in ethanolic media. In contrast to the granules formulated with lactose monohydrate, the presence of ethanol led to a significant reduction in drug release across all ethanol concentrations when compared to the ethanol-free control (f_2_ = 33.9 for 4% *v*/*v*, f_2_ = 31.1 for 20% *v*/*v*, and f_2_ = 40.2 for 40% *v*/*v*; *p* < 0.05). Interestingly, the inhibitory effect appeared slightly attenuated at the highest ethanol concentration, though still statistically significant.

This reduction in performance aligns with the known physicochemical properties of MCC. While MCC does not dissolve in either water or ethanol, its disintegrant function in aqueous media is mediated by its ability to swell and facilitate tablet breakup. Ethanol, however, has been reported to hinder this swelling process, thus delaying or even preventing the disintegration [55,71].

This interpretation was further corroborated by disintegration time measurements (Table 6). MCC-containing tablets disintegrated rapidly in ethanol-free medium, while increasing ethanol concentrations led to progressively extended disintegration times. At 40% ethanol, tablet disintegration was markedly delayed, providing direct evidence of inhibitory effect of ethanol on the swelling and disintegration capacity of MCC.

Collectively, these findings demonstrate that ethanol co-ingestion may introduce bidirectional risks for the sustained-release felodipine formulations investigated, with potential clinical consequences dependent on the type of filler incorporated. For wax–lactose monohydrate granules, while withstood ethanol concentrations up to 20% *v*/*v*, the pronounced acceleration in drug release under high-ethanol conditions still raises concerns regarding dose dumping. In the case of felodipine, which exhibits dose-dependent vasodilatory effects, excessive plasma concentrations resulting from unintended rapid release may lead to pronounced hypotension, reflex tachycardia, headache, and, in severe cases, vasodilatory shock, syncope or myocardial ischaemia [61,72]. Furthermore, individuals with alcohol dependence, who may consume substantial quantities of ethanol as part of their daily routine, represent a particular vulnerable population at heightened risk for adverse events. The severe overdose associated with dramatically increased drug release, nearly twofold in the present in vitro study, could precipitate life-threatening complications, including acute kidney injury [61].

Conversely, the ethanol-induced suppression of drug release from wax–MCC tablets, though mechanistically distinct, presents its own clinical concern. Inadequate drug release under ethanolic conditions could lead to subtherapeutic plasma concentrations, insufficient blood pressure control, and fluctuating pharmacodynamic responses. Given the chronic nature of hypertension management, such compromised drug availability may undermine long-term effectiveness of cardiovascular disease control [73].

Thus, these observations underscore the importance of comprehensive AIDD risk assessment, which extends beyond the classic concern of dose dumping to encompass ethanol–excipient interactions that may impair drug bioavailability. Careful excipient selection, coupled with rigorous in vitro stress testing that simulates worst-case ethanol exposure scenarios, remains critical essential for the development of reliable sustained-release dosage forms.

## 4. Conclusions

This study elucidated the formulation strategy for oral sustained-release felodipine dosage forms using wax matrices processed via HME. Syncrowax HGLC was demonstrated to be a viable wax carrier, enabling low-temperature processing, while MCC and lactose monohydrate served as fillers to facilitate smooth processing. All extruded tablets containing either filler exhibited acceptable friability (<1%), with tensile strength decreasing as the wax content increased.

MCC-containing tablets displayed superior wettability and disintegration properties, resulting in complete drug release over 24 h at optimal wax content (40% *w*/*w*). In contrast, lactose monohydrate tablets remained structurally intact during dissolution, limiting drug release; however, this was effectively mitigated by granulation, with 0.5–1 mm granules achieving favourable sustained-release profiles (up to 70% over 24 h).

Kinetic modelling revealed a shift towards Fickian diffusion with higher wax content for both filler-containing tablets, reflecting the increasing dominance of matrix diffusion in drug release. Ethanol susceptibility testing demonstrated distinct, filler-dependent behaviours: wax–MCC tablets suffered from release suppression across all ethanol concentrations, while wax–lactose monohydrate granules showed good tolerance up to 20% ethanol, with dose dumping observed at 40%. These bidirectional ethanol effects highlight that alcohol co-ingestion may not only accelerate drug release, risking dose dumping, but may also impede drug release by disrupting excipient functionality, potentially subtherapeutic exposure and compromised treatment efficacy.

In summary, this work underscores the pivotal role of wax level, filler selection, and formulation architecture in modulating the performance of wax-based sustained-release systems for felodipine, while providing valuable insights for the rational development of alcohol-resilient oral formulations. Future research can be expanded to include biorelevant dissolution testing, in vivo performance, and scale-up feasibility to explore the clinical translation potential of these formulations for long-term cardiovascular therapy.

## Figures and Tables

**Table 1:** HME processing temperature and screw speed for wax-based granule production.

Wax Level (*w*/*w*)	Felodipine (*w*/*w*)	Filler (Ratio *w*/*w*)	Process Temperature (°C)	Screw Speed (rpm)
40%	5%	MCC (55%)	70	100
50%	5%	MCC (45%)	67	100
60%	5%	MCC (35%)	65	100
30%	5%	Lactose monohydrate (65%)	66	120
40%	5%	Lactose monohydrate (55%)	65	100
50%	5%	Lactose monohydrate (45%)	64	80

**Table 2:** Physical properties of tablets prepared from wax–MCC and wax–lactose monohydrate extrudates (n = 6).

Filler Type	Wax Level (*w*/*w*)	Thickness (mm)	Density (g/cm^3^)	Breaking Force (N)	Tensile Strength (MPa)	Friability (%)
MCC	40%	2.41 ± 0.01	1.24 ± 0.005	71.4 ± 1.7	1.45 ± 0.03	0.28
	50%	2.53 ± 0.02	1.18 ± 0.007	70.2 ± 2.6	1.36 ± 0.04	0.30
	60%	2.65 ± 0.01	1.13 ± 0.004	62.5 ± 2.9	1.04 ± 0.05	0.39
Lactose	30%	2.29 ± 0.01	1.31 ± 0.005	66.0 ± 3.7	1.41 ± 0.08	0.39
	40%	2.39 ± 0.01	1.24 ± 0.004	62.2 ± 3.7	1.27 ± 0.08	0.18
	50%	2.52 ± 0.01	1.18 ± 0.004	51.7 ± 2.8	1.01 ± 0.05	0.15

**Table 3:** Korsmeyer–Peppas model parameters for dissolution profiles of wax-based tablets.

Wax Level (*w*/*w*)	MCC		Lactose Monohydrate	
	R^2^	*n*	R^2^	*n*
30%	-	-	0.999	0.67
40%	0.992	0.63	0.998	0.51
50%	0.970	0.59	0.990	0.39
60%	0.993	0.45	-	-

**Table 4:** Korsmeyer–Peppas model parameters for dissolution profiles of tested granules.

Wax Level (*w*/*w*)	Filler Type	Particle Size Range (mm)	R^2^	*n*
30%	Lactose monohydrate	0.5–1	0.994	0.51
30%	Lactose monohydrate	1–2	0.998	0.53
40%	MCC	0.5–1	0.998	0.46
40%	MCC	1–2	0.999	0.49
60%	MCC	0.5–1	0.995	0.46
60%	MCC	1–2	0.988	0.46

**Table 5:** Effect of ethanol concentration on the solubility of felodipine in the dissolution media (pH 6.5 PBS containing 2% *w*/*w* polysorbate 20) (n = 6).

Ethanol Concentration (% *v*/*v*)	Felodipine Solubility (mg/mL)
0	0.84 ± 0.03
4	0.86 ± 0.02
20	0.92 ± 0.05
40	3.24 ± 0.28

**Table 6:** Effect of ethanol on the disintegration time of 40% *w*/*w* wax–MCC tablets.

Ethanol Concentration (% *v*/*v*)	Disintegration Time (min)
0	5
4	9.5
20	11
40	18

## Data Availability

The raw data supporting the conclusions of this article will be made available by the authors on request.

## Supplementary Materials

The following supporting information can be downloaded at: https://www.mdpi.com/article/10.3390/pharmaceutics17080955/s1.
