# Supplementary material for: Wax-Based Sustained-Release Felodipine Oral Dosage Forms Manufactured Using Hot-Melt Extrusion and Their Resistance to Alcohol-Induced Dose Dumping
Source: Pharmaceutics. 2025 Jul 24;17(8):955. doi: 10.3390/pharmaceutics17080955 (PMC12388968; doi:10.3390/pharmaceutics17080955)
Supplement: Supplementary file 1 [file pharmaceutics-17-00955-s001.zip › pharmaceutics-3715249-supplementary.pdf]

Supplementary Materials for

Wax-Based Sustained-Release Felodipine Oral Dosage Forms Manufactured Using Hot-Melt Extrusion and Their Resistance to Alcohol-Induced Dose Dumping

Gerard Sweeney, Dijia Liu, Taher Hatahet, David S. Jones, Shu Li and Gavin P. Andrews *

*Correspondence to Prof. Gavin P. Andrews, E-mail: g.andrews@qub.ac.uk

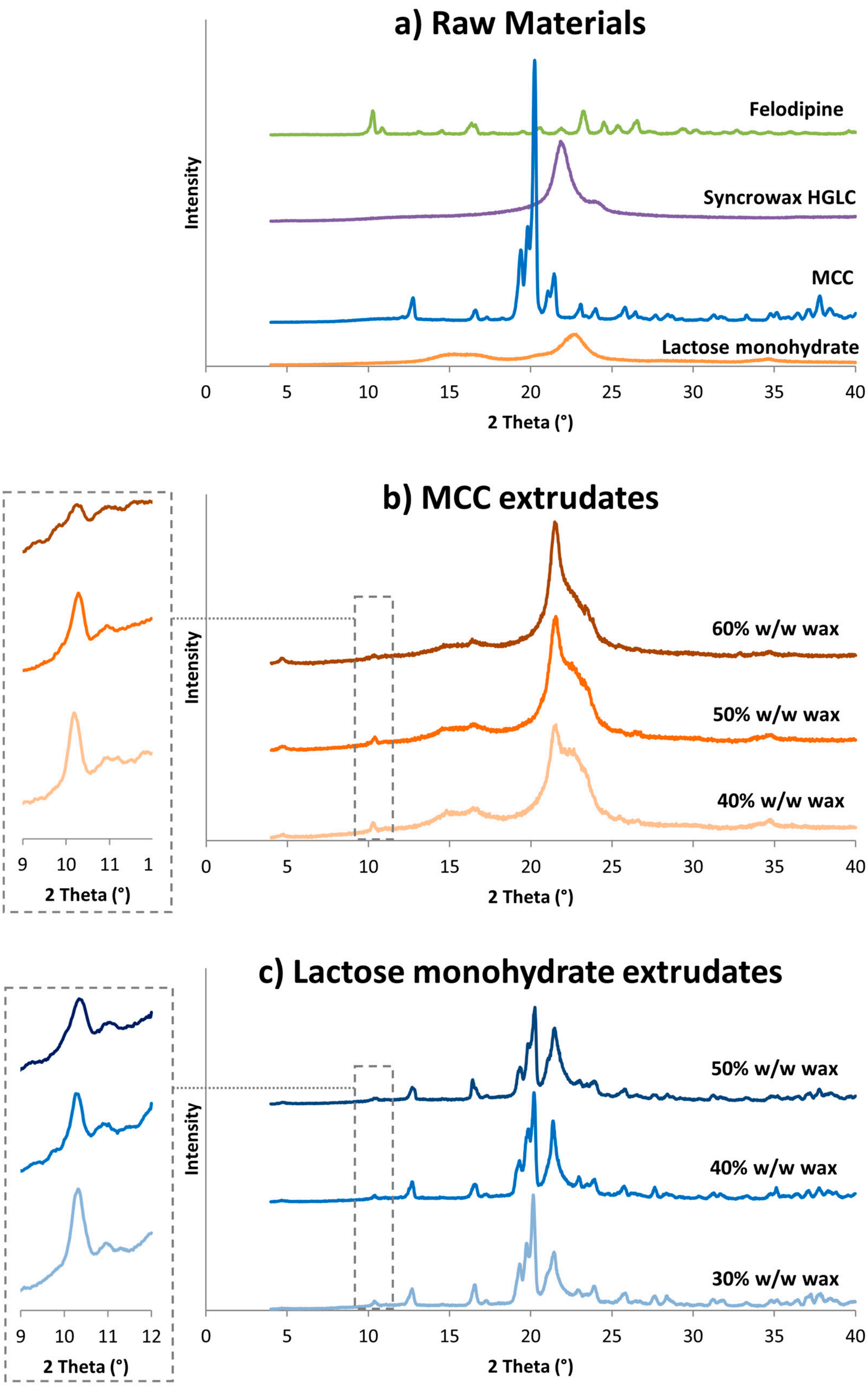

Figure S1. PXRD diffractograms of a) raw materials: felodipine (green), Syncrowax HGLC (purple), MCC (blue), and lactose monohydrate (orange); b) extrudates containing MCC as filler with 40%, 50%, and 60% w/w wax; and c) extrudates containing lactose monohydrate as filler with 30%, 40%, and 50% w/w wax. Insets show magnified regions of interest highlighting the characteristic diffraction peaks of crystalline felodipine.

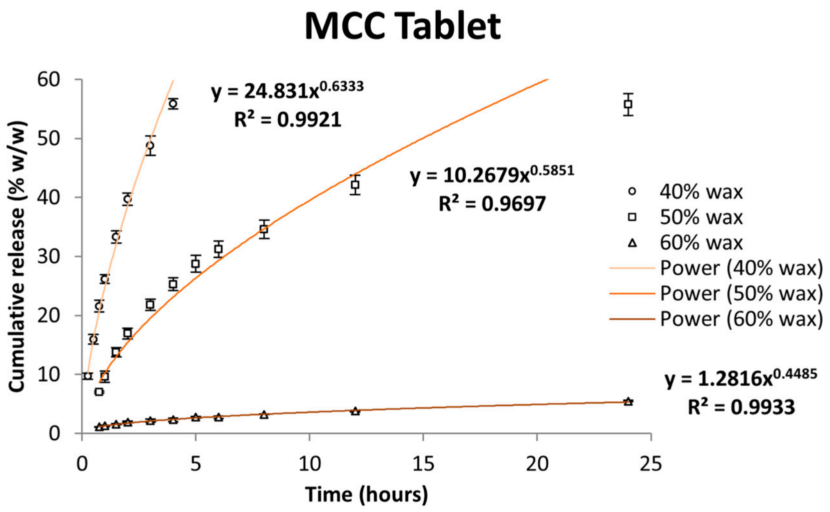
MCC Tablet
y = 24.831x^0.6333
R² = 0.9921
y = 10.2679x^0.5851
R² = 0.9697
y = 1.2816x^0.4485
R² = 0.9933
40% wax
50% wax
60% wax
Power (40% wax)
Power (50% wax)
Power (60% wax)
Cumulative release (% w/w)
Time (hours)
0
10
20
30
40
50
60
0
5
10
15
20
25

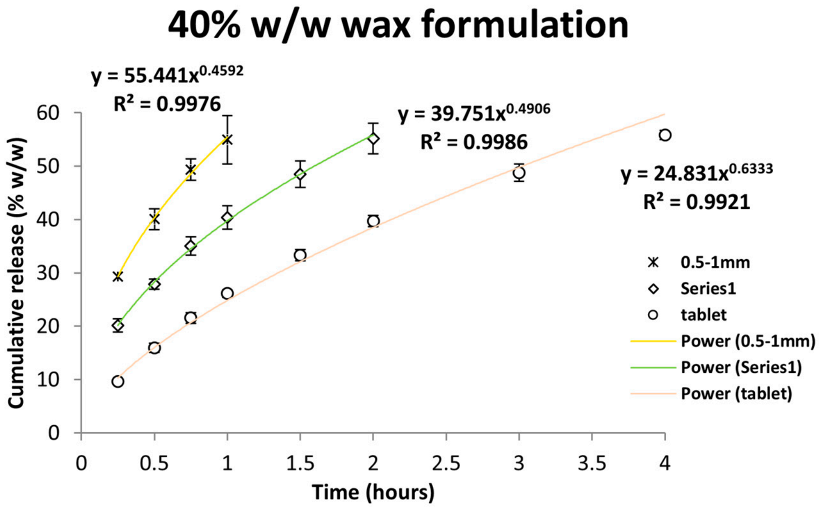
40% w/w wax formulation
y = 55.441x^0.4592
R² = 0.9976
y = 39.751x^0.4906
R² = 0.9986
y = 24.831x^0.6333
R² = 0.9921
0.5-1mm
Series1
tablet
Power (0.5-1mm)
Power (Series1)
Power (tablet)
Cumulative release (% w/w)
Time (hours)
0
10
20
30
40
50
60
0
0.5
1
1.5
2
2.5
3
3.5
4

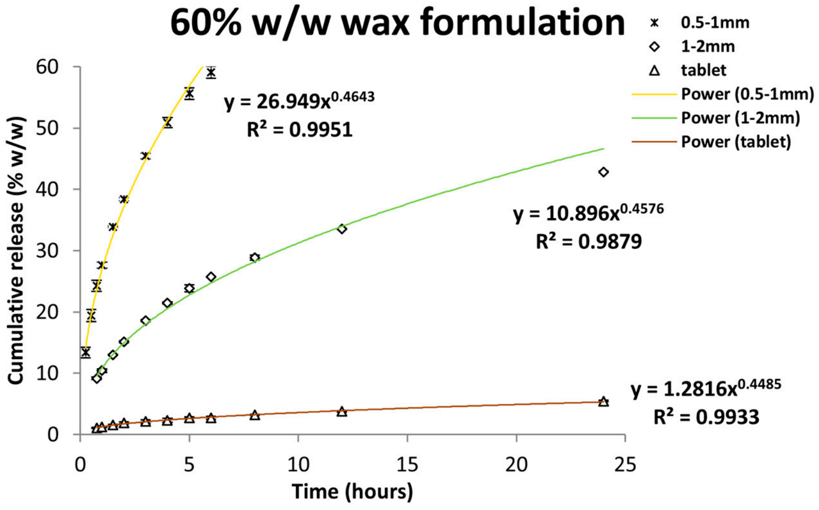
60% w/w wax formulation
0.5-1mm
1-2mm
tablet
Power (0.5-1mm)
Power (1-2mm)
Power (tablet)
y = 26.949x^0.4643
R² = 0.9951
y = 10.896x^0.4576
R² = 0.9879
y = 1.2816x^0.4485
R² = 0.9933
Cumulative release (% w/w)
Time (hours)
0
10
20
30
40
50
60
0
5
10
15
20
25

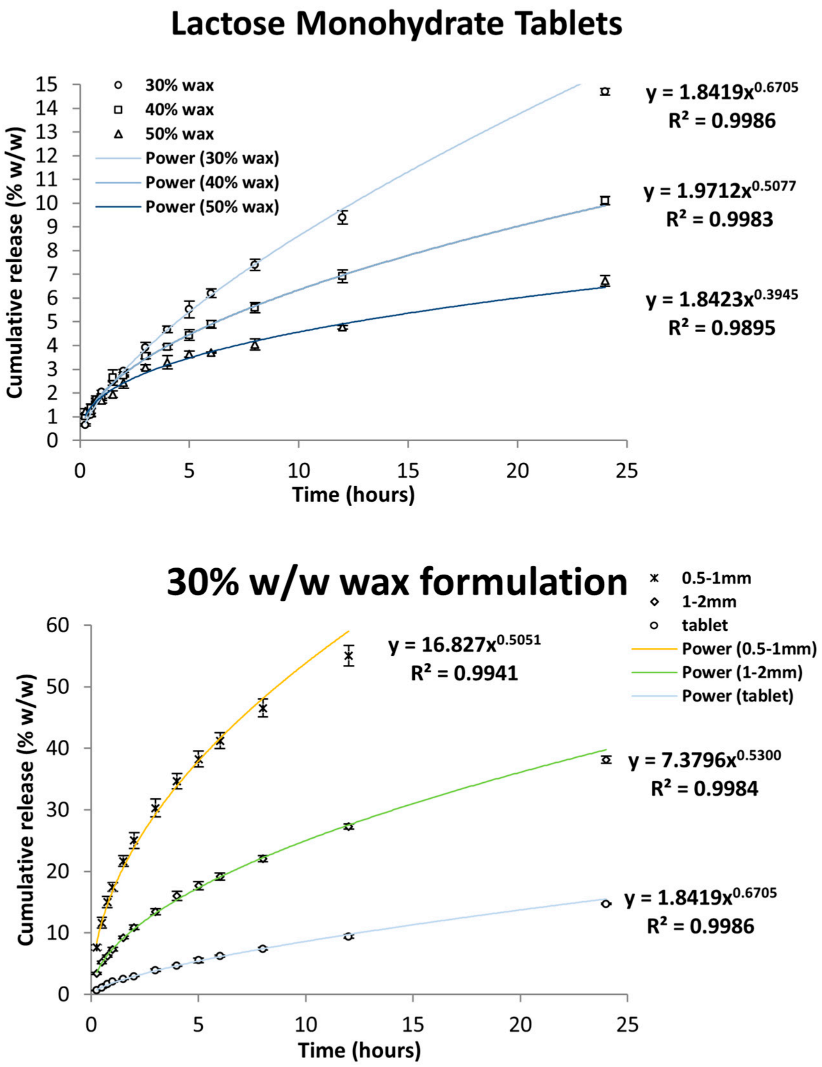

Figure S2. Korsmeyer–Peppas model fitting of felodipine release profiles, based on the portion of drug release below 60%. Experimental data are presented as symbols, with fitted curves shown as continuous lines. The corresponding equations and coefficients of determination (R^2) are provided alongside each dataset.
